# Comparative Analysis of Chemical Constituents in Peppers from Different Regions by Integrated LC-MS and GC-MS Non-Targeted Metabolomics

**DOI:** 10.3390/metabo16010085

**Published:** 2026-01-21

**Authors:** Xuefeng Gong, Sihao Hou, Yi Xu, Hong Li, Xin Chen, Zhanfeng Song

**Affiliations:** 1 Horticulture Research Institute, Sichuan Academy of Agricultural Sciences, Chengdu 610066, China; xuefenggong@scsaas.cn (X.G.); housh@scsaas.cn (S.H.);; 2 Horticultural Crops Germplasm Innovation and Utilization Key Laboratory of Sichuan Province, Chengdu 610066, China; 3 Key Laboratory of Horticultural Crops Biology and Germplasm Enhancement in Southwest Regions, Ministry of Agriculture and Rural Affairs, Chengdu 610066, China

**Keywords:** *Capsicum annuum*, metabolomics, fat, capsaicinoid, beta-alanine metabolism, differentially abundant metabolites (DAMs)

## Abstract

Background/Objectives: The quality of dried chili peppers is critically influenced by geographical origin, yet the metabolic basis for these differences remains insufficiently explored. This study sought to elucidate the region-specific metabolic profiles and their association with key quality traits in the pepper cultivar ‘Hong Guan 6’. Methods: Fruits harvested from three major cultivation regions in China were analyzed. We quantified fat and capsaicinoid content and employed an integrated LC-MS and GC-MS untargeted metabolomics approach to characterize the metabolite composition. Multivariate statistical analyses were applied to identify differentially abundant metabolites (DAMs) and uncover their related biochemical pathways. Results: Significant regional variations in fat and capsaicinoid content were observed, with peppers from Pengzhou (PZ) exhibiting the highest capsaicin levels. Metabolomic profiling revealed 529 metabolites that were significantly more abundant in PZ samples. These metabolites were enriched in several key pathways, including beta-alanine metabolism, plant hormone signal transduction, and N-glycan biosynthesis. Specifically, elevated levels of β-alanine and malonate in the beta-alanine metabolism pathway were detected in PZ and Anyue (AY) samples, suggesting a potential biochemical mechanism for their enhanced fat synthesis. Conclusions: Our findings demonstrate that geographical origin significantly reprograms the pepper metabolome, directly impacting quality attributes. The results provide crucial insights into the biochemical mechanisms, particularly those involving beta-alanine metabolism, that underpin the differences in critical quality traits such as fat content.

## 1. Introduction

Chili pepper (*Capsicum annuum* L.), originating from the tropical regions of Central and South America, has been used as a spice for over 8000 years [1,2]. It was not introduced to China until the late 16th century. Today, China has become the world’s largest producer and consumer, as well as a leading exporter of chili peppers, playing a pivotal role in the global market [3]. With a cultivation area exceeding two million hectares, numerous local varieties distinguished by their unique flavor and quality profiles have been developed [4]. Among these, dried chili peppers are particularly important in international trade due to their extended shelf life and market advantages [5].

The quality of dried chili peppers is primarily evaluated by attributes such as aroma and capsaicinoid content [6,7]. These traits are influenced not only by genetic factors (species and cultivars) but also significantly by environmental conditions and geographical origin [8,9]. Chili peppers contain a diverse array of metabolites, including terpenoids, acids, alcohols, esters, aldehydes, ketones, and phenolics. Among them, terpenoids, lipids, and aldehydes are major contributors to their distinct flavor characteristics [10,11,12,13]. The accumulation of these critical metabolites is profoundly shaped by local climatic conditions encompassing thermal regimes, precipitation patterns, and sunlight availability, all of which can reprogram metabolic pathways. For instance, studies on other crops, such as the spring maize–spring potato intercropping system in Pengzhou, have demonstrated that region-specific configurations of heat, water, and light resources are critical for coordinating growth and determining yield and quality traits [14]. Similarly, in pepper fruits, such climatic conditions lead to significant variations in the accumulation of key flavor and defense compounds, such as lipids and capsaicinoids.

Plant metabolite profiles are shaped by the interaction between genotype and environment. Environmental factors such as soil composition, temperature, and sunlight can trigger reprogramming of metabolic pathways, leading to variations in key flavor and defense compounds. Analyzing a single genotype across different environments, therefore, provides an effective strategy to isolate environmental effects on metabolite variation. Advanced metabolomics platforms, including ultra-performance liquid chromatography tandem mass spectrometry (UPLC-MS/MS) and integrated untargeted LC-MS/GC-MS approaches, have proven powerful in profiling such environmentally induced metabolic variation and identifying differentially expressed metabolites in *Capsicum* fruits [15,16]. Notably, such environment-induced metabolic variation is especially relevant in well-known production regions of China, including Guizhou and Sichuan, which are recognized for high-quality dried chili peppers [5,8]. For instance, a recent integrated metabolomic and transcriptomic study on the geographical indication pepper *‘Sichuan Erijingtiao’* revealed dynamic changes in volatile aroma compounds across developmental stages and identified a WRKY/bZIP-FAD/LOX regulatory module governing the biosynthesis of key aldehydes such as (E,E)-2,4-hexadienal [17]. These findings underscore the potential of multi-omics approaches to unravel the molecular mechanisms underlying aroma formation in peppers. Despite this reputation, the specific metabolite profiles underlying these regional quality differences remain poorly characterized.

To elucidate the metabolic basis of regional quality differences, we applied untargeted LC-MS and GC-MS metabolomics approaches to analyze the metabolite profiles of a single pepper cultivar cultivated in different regions. This study aims to decipher the variation in non-volatile metabolites influenced by growing region and to provide a theoretical foundation for understanding the regulatory networks underlying quality traits in dried chili peppers.

## 2. Materials and Methods

### 2.1. Plant Materials and Growth Conditions

The hot pepper (*Capsicum annuum* L.) cultivar ‘*Hong Guan 6*’ was sown on 10 January 2024, under natural conditions in a multi-span greenhouse in Xindu, Sichuan. Seedlings were grown for 12 weeks until transplanting. Then the seedlings were sent to three distinct geographic regions of China: Xiangchengxian, Henan (XX, 33.7258° N, 113.3131° E); Pengzhou, Sichuan (PZ, 30.9892° N, 103.9417° E); and Anyue, Sichuan (AY, 30.1039° N, 105.3361° E). The experimental design followed a randomized block design with six replicates per site. Transplanting to three open-field sites was conducted in early April, with planting on raised double-row beds at 40 cm spacing. Each experimental plot covered 333.35 m^2^ with six replicates. Uniform water and nutrient management were applied across all sites. Irrigation was performed via drip irrigation, with watering scheduled twice weekly based on soil moisture. Fertilization consisted of NPK compound fertilizer (15-15-15) applied at a rate of 300 kg/ha at transplanting. Fruits from the 4th to 6th nodes of main branches were harvested at 45 days post-anthesis (about 31 August 2025). Fresh samples (200 g) were dried at 40–60 °C to constant weight for subsequent analysis. For metabolomic analysis: Each of the six field plots per region was sampled independently—3 fruits were randomly selected from each plot to form one biological sample, resulting in 6 biological replicates per region (one replicate per field plot) for LC-MS/GC-MS detection. For quality trait analysis: A mixed sampling strategy was adopted to ensure representativeness—fruits from all six field plots per region were pooled, and three independent mixed subsamples were prepared from the pooled fruits, resulting in 3 technical replicates per region.

### 2.2. Analysis of Nutritional and Pungency Components

Pigment content was measured in accordance with the National Standard of China (GB 1886.34-2015) [18], and then the concentration was calculated based on the absorbance values. Fat content was determined gravimetrically following the Chinese National Standard (GB5009.6-2016) [19]. Briefly, 5 g of powdered sample was loaded into a cellulose thimble and subjected to Soxhlet extraction with anhydrous diethyl ether for 6–10 h at 60–80 °C. The solvent was subsequently evaporated, and the residual lipids were dried to a constant weight for quantification. Capsaicinoids, including capsaicin and dihydrocapsaicin, were analyzed based on the protocol outlined in GB/T 21266-2007 [20]. In brief, 5 g of dried pepper powder was accurately weighed and extracted ultrasonically three times (30 min each) with 25 mL of methanol-tetrahydrofuran (1:1, *v*/*v*) in a 60 °C water bath. After each extraction step, the mixture was filtered. The pooled filtrates were concentrated to 10–20 mL under reduced pressure at 70 °C and then made up to a final volume of 50 mL with the same extraction solvent for HPLC analysis. The daily maximum/minimum temperature for June to August 2024 from three planting sites was used for correlation analysis; the raw data were downloaded from https://www.tianqihoubao.com/lishi/ (accessed on 11 September 2025) and the C3S Climate Data Store ThermalTrace https://cds.climate.copernicus.eu/datasets (accessed on 31 October 2025).

### 2.3. Sample Preparation

For LC-MS analysis, metabolites were extracted from 30 mg of sample powder and aliquoted into a 1.5 mL tube. To each tube, we added two stainless-steel beads and 600 μL of ice-cold methanol–water (7:3, *v*/*v*) containing a mix of internal standards (4 μg/mL). The samples were pre-cooled at −40 °C for 2 min, then homogenized using a grinder (45 Hz, 2 min). Subsequent ultrasonication extraction was performed for 30 min in an ice-water bath. Following an overnight incubation at −40 °C, the extracts were centrifuged at 12,000 rpm for 20 min at 4 °C. A 150 μL aliquot of the resulting supernatant was passed through a 0.22 μm organic filter membrane into an LC vial and stored at −80 °C until analysis.

For GC-MS analysis, an additional derivatization step was employed. A 150 μL portion of the supernatant was transferred to a glass vial and completely dried using a centrifugal vacuum concentrator. The oximation reaction was carried out by adding 80 μL of methoxyamine hydrochloride (15 mg/mL in pyridine) and incubating at 37 °C for 60 min with shaking. Subsequently, a derivatization cocktail consisting of 50 μL BSTFA, 20 μL n-hexane, and 10 μL of a second internal standard mixture (C8-C24 FAMEs in chloroform) was added. The reaction proceeded at 70 °C for 60 min. After a 30-min cooling period to room temperature, the derivatized samples were analyzed by GC-MS.

### 2.4. LC-MS and GC-MS Metabolomic Profiling

LC-MS analysis was performed using a Waters ACQUITY UPLC I-Class plus/Thermo Scientific Q Exactive Plus Orbitrap Mass Spectrometer (Waters Corporation, Milford, MA, USA; Thermo Fisher Scientific Inc., Waltrham, MA, USA); separation was achieved on an ACQUITY UPLC HSS T3 column (100 mm × 2.1 mm, 1.8 μm) maintained at 45 °C. The mobile phase comprised (A) water with 0.1% formic acid (*v*/*v*) and (B) acetonitrile, delivered at 0.35 mL/min with a 3 μL injection volume. The gradient elution program was set as follows: 0–2 min, 5% B; 2–4 min, 5–30% B; 4–8 min, 30–50% B; 8–10 min, 50–80% B; 10–14 min, 80–100% B; 14–15 min, 100% B. Subsequently, the mobile phase composition was returned to the initial conditions (5% B) within 0.1 min (15–15.1 min) and maintained for 1 min (15.1–16 min) for column re-equilibration. GC-MS analysis was conducted on an Agilent 7890B-5977B (Agilent J&W Scientific, Santa Clara, CA, USA), a DB-5MS capillary column (30 m × 0.25 mm × 0.25 μm; Agilent J&W Scientific, Santa Clara, CA, USA) was employed under high-purity helium carrier gas (≥99.999%) at 1.0 mL/min. Samples (1 μL) were injected in splitless mode at 260 °C with a 6.2 min solvent delay. The oven temperature program initiated at 60 °C (0.5 min hold), followed by ramps at 8 °C/min to 125 °C, 8 °C/min to 210 °C, 15 °C/min to 270 °C, and 20 °C/min to 305 °C (5 min hold). Electron impact ionization (70 eV) was applied with source and quadrupole temperatures of 230 °C and 150 °C, respectively. Full-scan mass spectra were acquired across *m*/*z* 50–500.

### 2.5. Metabolite Data Analysis

Raw MS data were processed and annotated using a stringent multi-step pipeline. LC-MS data were processed with Progenesis QI (v3.0) for peak picking, alignment, and relative quantification. GC-MS data were processed with MS-DIAL (v4.24) for deconvolution and alignment. Metabolite identification was prioritized using plant-specific databases (the in-house LuMet-Plant 3.0 and LuMet-GC 5.0 databases) to enhance biological relevance, supplemented by public databases (METLIN). For LC-MS features, putative identifications required matching of both precursor mass (tolerance: 10 ppm) and MS/MS fragmentation spectra (tolerance: 20 ppm). Identifications were assigned a confidence level (Levels 1–4); only those with high confidence (Levels 1 & 2, corresponding to matches with authentic standards or high-quality spectral libraries) were retained for downstream analysis. The final data matrix underwent rigorous filtering (RSD < 30%, missing value imputation) and was log2-transformed, mean-centered, and subjected to Pareto scaling. Multivariate statistical analysis and unsupervised principal component analysis (PCA) were performed using the R package gmodels (v2.18.1) (https://github.com/r-gregmisc/gmodels, accessed on 2 June 2025). A supervised partial least squares discrimination analysis and orthogonal partial least squares discrimination analysis (OPLS-DA) were then conducted to distinguish the overall differences in metabolic profiles between groups and identify their differential metabolites. Each supervised model was validated through 7-fold cross-validation and 200 permutation tests. Additionally, a cross-validated analysis of variance was performed for each model to assess its statistical significance. The criteria for screening differentially abundant metabolites (DAMs) between the groups were identified based on the variable importance in the projection (VIP) value ≥ 1, *p*-value < 0.05, and FC ≥ 4.0 or FC ≥ 1/4.0 [21]. The DAMs were analyzed for Kyoto Encyclopedia of Genes and Genomes (KEGG, https://www.kegg.jp/, accessed on 2 June 2025) pathway enrichment analysis using a hypergeometric test [22]. Statistical significance was assessed using a two-tailed Student’s *t*-test. To control the false discovery rate in multiple comparisons, the adjusted Benjamini–Hochberg (BH) *q*-values < 0.05 were considered significant for differential metabolite screening, correlation analyses, and KEGG pathway enrichment.

## 3. Results

### 3.1. Physicochemical Characteristics of Peppers in Three Regions

**Figure 1:**
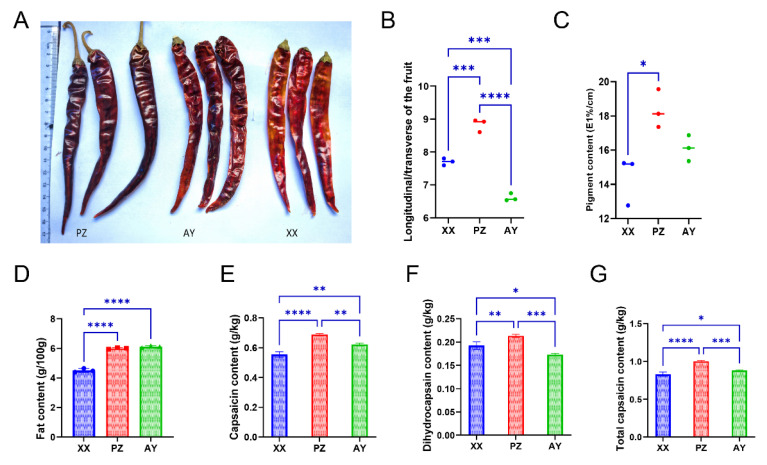
Phenotypic and biochemical variations in peppers from different growing regions. (**A**) Fruit morphology. (**B**) Fruit index. (**C**) Pigment content. (**D**) Fat content. (**E**) Capsaicin content. (**F**) Dihydrocapsaicin content. (**G**) Total capsaicinoid content. XX, Xiangxian; PZ, Pengzhou; AY, Anyue. Data points represent mean ± SD (*n* = 3). Asterisks (*) indicate significant differences (*, *p* < 0.05; **, *p* < 0.01; ***, *p* < 0.001; ****, *p* < 0.0001).

We compared the fruits from three cultivation regions and found no remarkable morphological differences among them. However, peppers from the PZ origin exhibited a deeper red color (Figure 1A). Fruits from PZ showed a higher fruit length/diameter ratio (Figure 1B). Analysis of pigment content revealed that PZ samples had the highest levels, which were significantly greater than those from XX (Figure 1C). Analysis of key quality indicators revealed that the fat content was lowest in samples from XX (4.5 ± 0.07 g/100 g), while those from PZ and AY contained approximately 6.0 ± 0.03 g/100 g and 6.1 ± 0.04 g/100 g, which were significantly higher than that of XX (Figure 1D). Capsaicin content measurements showed significant differences among all three origins, with PZ (0.687 ± 0.004 g/Kg) displaying markedly higher levels than AY (0.621 ± 0.005 g/Kg) and XX (0.554 ± 0.01 g/Kg) (Figure 1E–G). Dihydrocapsaicin content was 0.192 ± 0.004 g/Kg (XX), 0.213 ± 0.002 g/Kg (PZ), and 0.173 ± 0.001 g/Kg (AY). Total capsaicinoid content was 0.829 ± 0.017 g/Kg (XX), 1.0 ± 0.007 g/Kg (PZ), and 0.882 ± 0.004 g/Kg (AY) (Figure 1E,F).

### 3.2. Environmental Factors Related to Physicochemical Characteristics of Peppers in Three Regions

**Figure 2:**
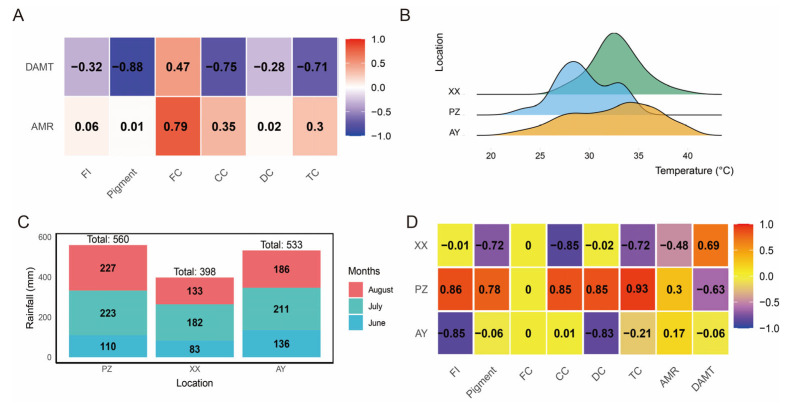
Correlation analysis of environmental factors. (**A**) Correlation analysis of DAMT and AMR with physicochemical characteristics. (**B**) Daily maximum temperature from June to August. (**C**) Rainfall from June to August. (**D**) Correlation analysis of samples with physicochemical characteristics and environmental factors. DAMT, daily maximum average temperature; AMR, Average monthly rainfall; FI, Fruit index; FC, Fat content; CC, Capsaicin content; DC, Dihydrocapsaicin content; TC, Total capsaicinoid content. Note: Correlation analyses are based on mean values from the three cultivation sites. They are presented for descriptive purposes only to illustrate potential environmental associations.

Furthermore, we found that the daily maximum temperature was negatively correlated with pigment content, capsaicin content, and total capsaicin content, with correlation coefficients exceeding 70%. In contrast, the monthly average rainfall was positively correlated with fat content, showing a correlation of 79% (Figure 2A). Analysis of the daily maximum temperature distribution from June to August across the three sites showed that temperatures at the PZ site were predominantly around 28 °C, whereas at the XX site they clustered near 34 °C. Although the AY site also reached around 35 °C, the distribution was relatively even across the 28–35 °C range without a dominant concentration (Figure 2B). In terms of accumulated rainfall during the same period, the XX site received the least precipitation, while both the AY and PZ sites exceeded 500 mm (Figure 2C). Correlation analysis between samples, environmental factors, and biochemical traits revealed that the PZ sample was positively correlated with fruit index (FI), pigment, and capsaicin content (all above 78%), but negatively correlated with daily maximum temperature (−63%). In contrast, the XX sample showed positive correlation (69%) with daily maximum temperature (Figure 2D).

### 3.3. Metabolite Identification

**Figure 3:**
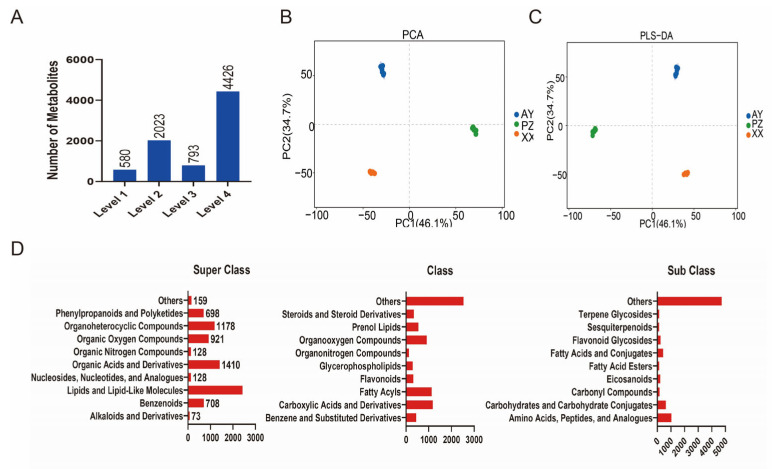
Identification and data analysis of Metabolites. (**A**) Statistics of metabolite quantity. (**B**) PCA of the LC/GC-MS data. (**C**) PLS-DA of the LC/GC-MS data. (**D**) The superclass, class and sub class of 7822 metabolites identified.

A total of 7822 metabolites were identified, following rigorous filtering (retaining features with CV < 30% in QC samples, removing background signals, and consolidating adducts), and applying the identification criteria described above, the final dataset for statistical analysis consisted of 2603 annotated metabolites with high confidence (Levels 1 & 2) (Figure 3A, Supplementary Table S1). Principal Component Analysis (PCA) revealed clear separation among the three sample groups, indicating significant differences in metabolite profiles based on geographical origin (Figure 3B). Further analysis using Partial Least Squares-Discriminant Analysis (PLS-DA) confirmed pronounced compositional differences between groups (Figure 3C).

According to the HMDB classification system at the superclass level, the major categories of metabolites included Lipids and lipid-like molecules (2419, 30.93%), Organic Acids and Derivatives (1410, 18.03%), Organic Oxygen Compounds (921, 11.77%), and Organoheterocyclic Compounds (1178, 15.06%). At the class level, the largest proportion was classified as “Others”; other predominant classes comprised Carboxylic Acids and Derivatives (1181, 15.10%), Fatty Acyls (1122, 14.34%), and Organooxygen Compounds (911, 11.65%). Further subdivision at the subclass level indicated that the main metabolic constituents were Amino Acids, Peptides, and Analogues (1030, 13.17%), Carbohydrates and Carbohydrate Conjugates (631, 8.07%), and Fatty Acids and Conjugates (422, 5.40%) (Figure 3D).

**Figure 4:**
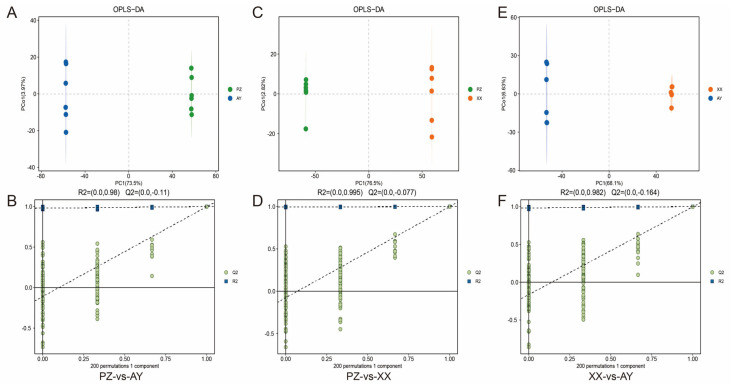
OPLS−DA of the LC/GC–MS data and validation. (**A**) OPLS−DA of the PZ-vs-AY data. (**B**) Response permutation testing of PZ-vs-AY data. (**C**) OPLS−DA of the PZ-vs-XX data. (**D**) Response permutation testing of PZ-vs-XX data. (**E**) OPLS−DA of the XX-vs-AY data. (**F**) Response permutation testing of XX-vs-AY data. R2X (cum): cumulative interpretation rate in the X direction; R2Y (cum): cumulative interpretation rate in the Y direction; Q2 (cum): cumulative forecast rate of the model; R2 and Q2: parameters of the response sequencing test used to measure whether the model was overfitted. XX, Xiangxian; PZ, Pengzhou; AY, Anyue.

Furthermore, we employed OPLS-DA to maximize the separation between the two sample groups. The score plot demonstrated clear discrimination among the groups (Figure 4A,C,E). Permutation testing results indicated an R2 value of approximately 0.99. Additionally, the green regression line of the Q2 points intersected the vertical axis (left side) below zero, confirming that the model was robust and reliable (Figure 4B,D,F).

### 3.4. DAMs Identification and Function Enrichment

**Figure 5:**
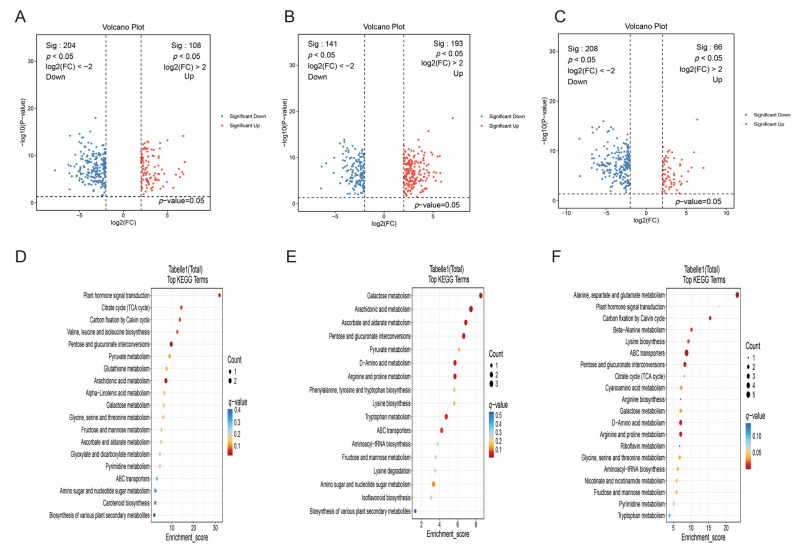
Screening and functional enrichment analysis of DAMs. (**A**) Volcano plot of DAMs in the PZ vs. AY comparison. (**B**) Volcano plot of DAMs in the PZ vs. XX comparison. (**C**) Volcano plot of DAMs in the XX vs. AY comparison. (**D**) Enriched KEGG pathways of DAMs in the PZ vs. AY group. (**E**) Enriched KEGG pathways of DAMs in the PZ vs. XX group. (**F**) Enriched KEGG pathways of DAMs in the XX vs. AY group. DAMs were identified based on VIP ≥ 1, |FC| ≥ 4, and BH-adjusted *q*-value < 0.05.

To investigate the quality differences in peppers cultivated in different regions, we compared the DAMs between each pair of groups. The results revealed that in the PZ vs. AY comparison, 438 metabolites were significantly up-regulated and 596 were significantly down-regulated. Of these, 108 up-regulated (e.g., Aristolene (VIP = 1.6, FC = 4.7, q = 6.1 × 10^−11^), 9-hydroxy pelargonic acid (VIP = 1.9, FC = 9.3, q = 3.7 × 10^−10^)) and 204 down-regulated (e.g., PC(18:4/0:0) (VIP = 2.6, FC = 0.01, q = 2.4 × 10^−12^), Dehydrocurdione (VIP = 2.1, FC = 0.06, q = 3.1 × 10^−11^)) DAMs were assigned high-confidence annotations (levels 1–2) (Figure 5A, Supplementary Table S2). In the PZ vs. XX comparison, 636 metabolites were up-regulated and 484 were down-regulated. Among these, 193 and 141 DAMs were identified as high-confidence (levels 1–2) up- (e.g., 3s,4-Dihydroxybutyric Acid (VIP = 1.5, FC = 4.1, q = 4.4 × 10^−11^), N2-Maltulosylarginine (VIP = 1.7, FC = 6.8, q = 2.6 × 10^−10^)) and down-regulated (e.g., 19S-HETE (VIP = 1.7, FC = 0.12, q = 1.4 × 10^−10^), Abscisic alcohol 11-glucoside (VIP = 1.6, FC = 0.2, q = 6.8 × 10^−10^)) metabolites, respectively (Figure 5B, Supplementary Table S3). For the XX vs. AY comparison, 269 metabolites were up-regulated and 561 were down-regulated. Within this set, 66 were up-regulated (e.g., 1-O-p-Coumaroyl-(b-D-glucose 6-O-sulfate) (VIP = 1.7, FC = 4.6, q = 7.9 × 10^−8^), D-Sorbitol (VIP = 1.6, FC = 4.2, q = 3.0 × 10^−7^)) and 208 were down-regulated (e.g., PC(17:2/0:0) (VIP = 1.7, FC = 0.19, q = 1.8 × 10^−14^); Ile-Val (VIP = 1.7, FC = 0.2, q = 6.5 × 10^−12^)) DAMs corresponded to high-confidence annotations (levels 1–2) (Figure 5C, Supplementary Table S4).

Further functional enrichment analysis of these DAMs indicated that in the PZ vs. AY group, the metabolites were primarily enriched in pathways such as plant hormone signal transduction (Figure 5D). The DAMs in the PZ vs. XX group were mainly associated with Galactose metabolism, Arachidonic acid metabolism, and so on (Figure 5E). In the XX vs. AY comparison, significant enrichment was observed in pathways including alanine, aspartate and glutamate metabolism, plant hormone signal transduction, beta-alanine metabolism, and so on (Figure 5F).

### 3.5. DAMs Associated with Different Regions

**Figure 6:**
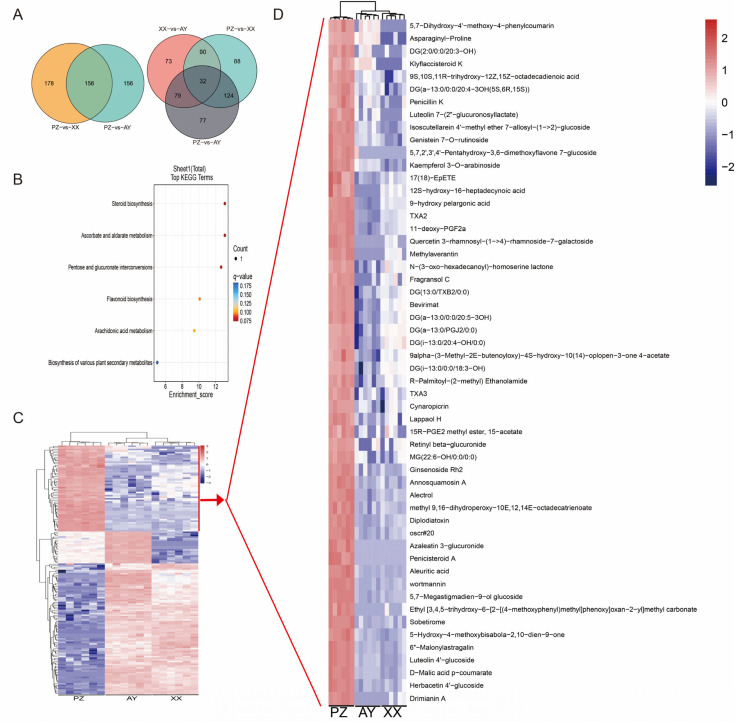
DAMs screening and heatmap of metabolite abundance. (**A**) Venn diagram of the DAMs. (**B**) KEGG pathways enrichment of the interaction DAMs between PZ vs. AY and PZ vs. XX comparison groups. (**C**) Heatmap of 156 interaction DAMs. (**D**) Heatmap of the cluster1 DAMs. The color scale is shown in the right area. *q*-value < 0.05.

Measurement of capsaicin content revealed significantly higher levels in the PZ samples compared to those from the other two cultivation regions. We identified 156 common DAMs between the PZ vs. AY and PZ vs. XX comparison groups (Figure 6A), which may be associated with the higher capsaicin content in the PZ samples. Furthermore, analysis of group-specific DAMs showed that the PZ vs. AY, PZ vs. XX, and XX vs. AY comparisons contained 7788, and 73 unique DAMs, respectively (Figure 6A). These unique DAMs may contribute to differences in capsaicin composition among the regions. Enrichment analysis revealed that 156 overlapping DAMs between the PZ vs. AY and PZ vs. XX comparison groups were significantly enriched in pathways such as Steroid biosynthesis (Figure 6B).

Metabolite abundance analysis of the 156 common DAMs shared between the PZ vs. AY and PZ vs. XX groups demonstrated significant regional variations. Based on their accumulation patterns, the DAMs were classified into three major subclusters. Notably, metabolites in cluster1 were significantly down-regulated in PZ samples, whereas those in cluster1 were significantly up-regulated (Figure 6C,D). Whether these up-regulated DAMs are functionally linked to the high capsaicin content in PZ samples warrants further investigation.

### 3.6. Beta-Alanine Metabolism Pathway

**Figure 7:**
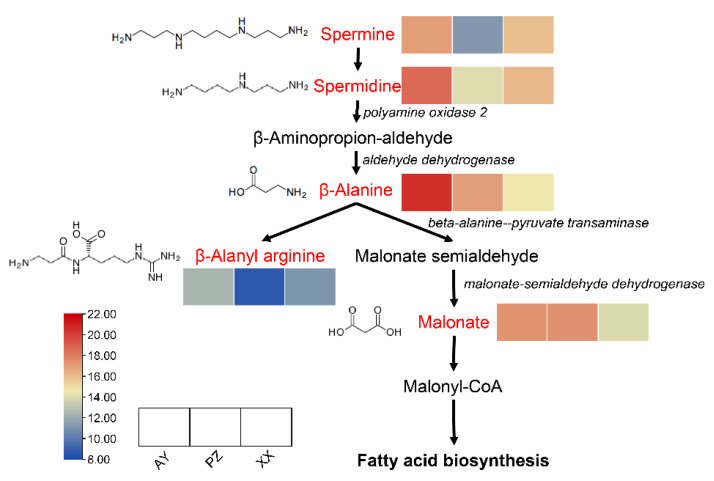
Visualization of the metabolite dynamics in the beta-alanine metabolism pathway map. The red letter shows the enrichment metabolites. The italic letter shows key genes. Within each box, the rows represent the different samples (from left to right). Each metabolite intensity was performed using a heatmap, and the color scale is shown below.

As indicated by the earlier results, DAMs from multiple comparison groups were significantly enriched in the beta-alanine metabolism pathway. Analysis of the metabolites enriched in this pathway revealed a total of five metabolites that showed significant differences among the sample groups. Among them, the contents of spermine, spermidine, and β-alanyl arginine were lower in the PZ samples than in the AY and XX samples. In contrast, the levels of β-alanine and malonate were higher in both AY and PZ samples compared to the XX samples. This pattern may be associated with the higher lipid content observed in the AY and PZ samples (Figure 7).

### 3.7. Plant Hormone Signal Transduction and N-Glycan Biosynthesis Pathway

**Figure 8:**
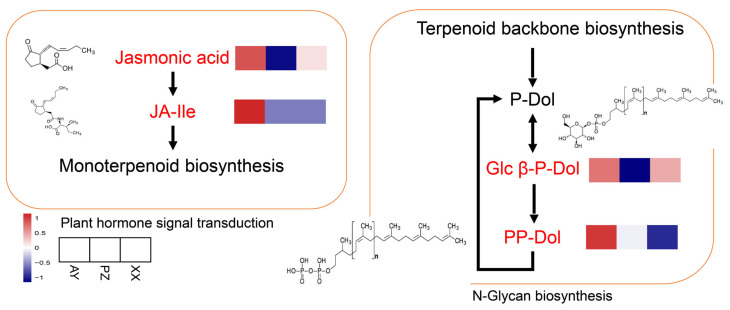
Visualization of the metabolite dynamics in plant hormone signal transduction and N-Glycan biosynthesis pathway. The red letter shows the enrichment metabolites. Within each box, the rows represent the different samples (from **left** to **right**). Each metabolite intensity was performed using a heatmap, and the color scale is shown below.

Additionally, we found that the Plant hormone signal transduction and N-Glycan biosynthesis pathways were also significantly enriched with DAMs. Within the plant hormone signal transduction pathway, significant enrichment was observed only in the Jasmonate-related hormonal pathway. The abundance of Jasmonic acid was relatively higher in the AY and XX samples and lowest in the PZ samples. Meanwhile, the downstream metabolite JA-Ile exhibited the highest abundance in AY samples and was relatively lower in both PZ and XX samples. This pattern suggests a potential influence on the downstream Monoterpenoid biosynthesis. In the N-Glycan biosynthesis pathway, two metabolites also showed significant abundance variations. Specifically, Glcβ-P-Dol was more abundant in AY and XX samples compared to PZ, whereas PP-Dol reached its highest abundance in AY samples and was lower in both PZ and XX samples. The abundance of metabolites in the N-Glycan biosynthesis pathway is known to depend on key products from the upstream Terpenoid backbone biosynthesis pathway (Figure 8).

## 4. Discussion

As an important culinary ingredient, pepper (*Capsicum*) is not only rich in nutrients but also has been widely used in medicine for its antibacterial and antioxidant properties [23,24,25,26]. Extensive studies have shown that factors such as geographic region, environment, climate, and cultivar can significantly affect pepper quality [2,3,5,7,8]. The quality of peppers is largely determined by the composition and abundance of various metabolites. To investigate the metabolic differences in peppers grown in different regions, this study compared the metabolomes of the same pepper variety cultivated in Sichuan and Henan provinces.

We found that the peppers from the three cultivation sites not only showed distinct differences in color but also exhibited noticeable variations in the fruit length-to-width ratio (fruit morphology and fruit index). The lipid content was significantly higher in the two Sichuan samples than in the Henan samples. The two Sichuan samples were geographically closer in latitude, suggesting potentially similar sunlight exposure and soil conditions (Figure 1). Furthermore, malonyl-CoA is a direct product of the beta-alanine metabolism pathway and a precursor for fatty acid synthesis [27], showed more abundance in the Sichuan samples (PZ and AY). Consistent with this, both beta-alanine and malonate were enriched in the PZ and AY samples, providing indirect evidence supporting their higher lipid content (Figure 6). However, capsaicinoid biosynthesis relies on distinct precursors from the phenylpropanoid (vanillylamine) and branched-chain amino acid pathways, which were not highlighted in our untargeted data. Thus, while beta-alanine metabolism may contribute to lipid accumulation, it does not provide a mechanistic basis for the observed differences in capsaicinoid content.

The findings from Jeeatid et al. indicate significant differences among variety, environment, and their interaction [28]. The environmental factor effect accounted for 67.7%, while the genotypic effect accounted for 42.4%. Higher levels of capsaicinoids were produced under conditions with higher relative humidity and lower light intensity [28]. Contrary to several studies reporting enhanced capsaicinoid biosynthesis under higher temperatures (25–37 °C), our results indicated a significant negative correlation between daily maximum temperature and capsaicinoid content (Figure 2A,D). The coolest site (PZ, ~28 °C) exhibited the highest capsaicinoid levels, whereas the warmer XX site (~34 °C) showed the lowest. This apparent contradiction may be explained by the unique combined climatic factors in the Sichuan Basin cultivation sites (PZ and AY), where high relative humidity and lower light intensity—factors previously linked to higher capsaicinoid levels—predominate. Furthermore, the response to temperature may be cultivar-specific in ‘Hong Guan 6’, suggesting that optimal capsaicinoid accumulation might occur under a narrower or lower temperature range in this genotype. Future studies controlling for humidity and light independently are needed to dissect these interacting environmental effects.

Terpenoids are important volatile compounds responsible for the aroma in chili peppers. While jasmonate is known to be involved in the biosynthesis of monoterpenoids, studies have also shown that it regulates capsaicinoid synthesis by inducing MYB-type transcription factors [29,30]. In this study, we found that the endogenous level of JA-Ile, the primary bioactive form of jasmonate, was highest in the AY samples but relatively low in the PZ samples, which exhibited high capsaicinoid content. This inverse correlation suggests that the relationship may be more complex than a simple linear activation. Potential explanations include temporal decoupling of JA-Ile signaling and capsaicinoid accumulation peaks, negative feedback mechanisms at high capsaicinoid levels, or the involvement of alternative signaling pathways or jasmonate metabolites in this specific cultivar and environment. Additionally, N-Glycan biosynthesis is a downstream pathway that shares the terpenoid backbone precursors. Our results also showed that the abundance of Glcβ-P-Dol and PP-Dol metabolites was relatively lower in both PZ and XX samples. This may suggest a reduced flux through the upstream terpenoid backbone pathway in these samples, potentially diverting resources away from certain downstream branches. Beyond the differences in capsaicinoid content measured in this study, the volatile aroma profiles of these samples remain to be analyzed. The potential variation in these compounds may be associated with their geographic origins.

## 5. Conclusions

Our study revealed significant differences in lipid and capsaicin content in the same variety of chili peppers grown in different regions. To elucidate the impact of these variations on fruit quality, we employed a metabolomics approach and identified 529 metabolites that were significantly more abundant in PZ samples compared to AY and XX samples. These metabolites were primarily associated with pathways such as beta-alanine metabolism, plant hormone signal transduction, and N-glycan biosynthesis. Specifically, differences in the abundance of β-alanine and malonate within the beta-alanine metabolism pathway may contribute to enhanced fat synthesis, thereby increasing lipid levels in PZ and AY samples. Additionally, jasmonate may play a role in both monoterpenoid biosynthesis and the regulation of capsaicin accumulation. Future research should analyze volatile aroma compounds to complete the metabolic profile and experimentally validate whether these components directly cause the regional quality differences.

## Data Availability

The raw and processed metabolomics data are publicly available in China National Center for Bioinformation (CNCB) with the accession number PRJCA053697 (https://ngdc.cncb.ac.cn/bioproject/browse/PRJCA053697, accessed on 15 December 2025).

## Supplementary Materials

The following supporting information can be downloaded at: https://www.mdpi.com/article/10.3390/metabo16010085/s1, Table S1: The metabolite abundance data; Table S2: The DAM list of PZ vs. AY groups; Table S3: The DAM list of PZ vs. XX groups; Table S4: The DAM list of XX vs. AY groups.

## Abbreviations

The following abbreviations are used in this manuscript:

DAMs	Differentially abundant metabolites
PZ	Pengzhou city (Sichuan Province)
AY	Anyue county (Sichuan Province)
XX	Xiangcheng county (Henan Province)
